# Curcumin-loaded mesoporous silica nanoparticles for drug delivery: synthesis, biological assays and therapeutic potential – a review

**DOI:** 10.1039/d3ra02772d

**Published:** 2023-07-24

**Authors:** Milad Iranshahy, Mohammad Yahya Hanafi-Bojd, Seyed Hadi Aghili, Mehrdad Iranshahi, Seyed Mohammad Nabavi, Satar Saberi, Rosanna Filosa, Iman Farzam Nezhad, Maede Hasanpour

**Affiliations:** a Department of Pharmacognosy, School of Pharmacy, Mashhad University of Medical Sciences Mashhad Iran; b Nanomedicine Department, Faculty of Medicine, Birjand University of Medical Sciences Birjand Iran; c Valiasr Hospital, Department of Neurosurgery Tehran Iran; d Biotechnology Research Center, Pharmaceutical Technology Institute, Mashhad University of Medical Sciences Mashhad Iran Hasanpourmm3@mums.ac.ir maede.hasanpour@yahoo.com; e Advanced Medical Pharma (AMP-Biotec), Biopharmaceutical Innovation Centre Via Cortenocera 82030 San Salvatore Telesino BN Italy; f Nutringredientes Research Center, Federal Institute of Education, Science and Technology (IFCE) Brazil; g Department of Chemistry, Faculty of Science, Farhangian University Tehran Iran; h Dipartimento di Scienze e Tecnologie, Università Degli Studi Del Sannio Benevento Italy; i Department of Chemistry, Faculty of Sciences, Ferdowsi University of Mashhad Mashhad Iran

## Abstract

Curcumin-loaded mesoporous silica nanoparticles (MSNs) have shown promise as drug delivery systems to address the limited pharmacokinetic characteristics of curcumin. Functionalization with folic acid and PEGylation enhance anticancer activity, biocompatibility, stability, and permeability. Co-delivery with other drugs results in synergistically enhanced cytotoxic activity. Environment-responsive MSNs prevent undesirable drug leakage and increase selectivity towards target tissues. This review summarizes the methods of Cur-loaded MSN synthesis and functionalization and their application in various diseases, and also highlights the potential of Cur-loaded MSNs as a promising drug delivery system.

## Abstract 2

Curcumin-loaded mesoporous silica nanoparticles (MSNs) have shown promise as drug delivery systems to address the limited pharmacokinetic characteristics of curcumin.

## Graphical abstract

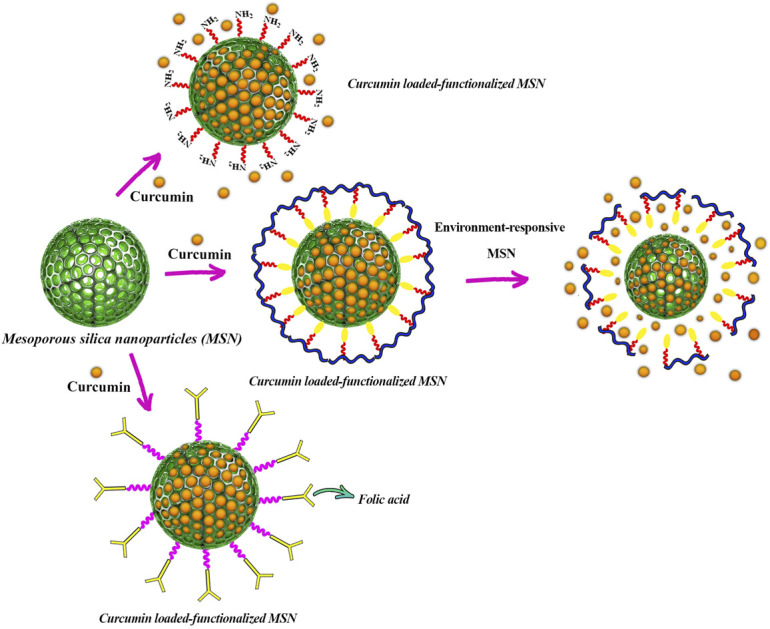

## Introduction

**Fig. 1:**
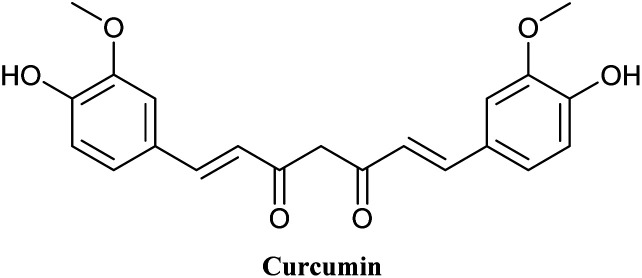
Chemical structure of curcumin.

Curcumin (Cur) is a natural polyphenol compound isolated from the rhizomes of *Curcuma longa* (turmeric) that acts as a high-potency bioactive agent to treat a variety of diseases such as diabetes, cancer, arthritis and neurological diseases^1^ (Fig. 1). The therapeutic effects of Cur are mostly attributable to its anti-inflammation, antioxidant, and especially anticarcinogenic activities. Cur has been used successfully in the prevention of clinical cancer, especially breast cancer.^2,3^ Recently, a clinical trial study was conducted on patients with advanced and metastatic breast cancer to evaluate the safety and efficacy of Cur in combination with paclitaxel.^4^ In fact, Cur suppresses the growth of cancer cells by inducing the production of reactive oxygen species (ROS) and increasing apoptosis in cancer cells.^5,6^ Cur exhibited high safety profile in a variety of animal and human studies, even at very high doses.^7–10^ The intrinsic allochroic effect and fluorescence properties of Cur allow it to act as a superior fluorescent marker for distribution in cells and tissues for the detection of biomarkers, fluorescence imaging and dual-signal reporting.^11–13^ Cur possesses theranostic properties that can be used simultaneously in cancer targeting, diagnosis, and treatment.^14^

However, the therapeutic potential of Cur is restricted due to its undesirable physicochemical and pharmacokinetic characteristics. Cur is practically water-insoluble (11 ng mL^−1^) at acidic and neutral pH and soluble in alkali condition. It is also chemically unstable at physiological conditions with a half-life (*t*_1/2_) of less than 10 min in buffer solution (pH = 7.4). These challenges, as well as the poor bioavailability of Cur, are the main limitations of its therapeutic applications.^1,15–17^ Various efforts have been focused on enhancing its poor water solubility and undesirable pharmacokinetic characteristics. Several nanoparticulate drug delivery techniques including liposomes^18,19^ nanoemulsions,^20,21^ polymeric nanoparticles^22^ and nanosuspensions,^23^ have been harnessed to tackle this problem.

In recent decades, mesoporous silica nanoparticles (MSNs) have attracted the interest of many researchers as a promising drug carrier for therapeutic applications due to their unique properties.^24–27^ MSNs have tunable pore sizes ranging from 2 to 50 nm in diameter as well as a high loading capacity with a pore volume of greater than 0.6 cm^3^ g^−1^, making them a suitable agent as drug carriers and dissolution enhancement. Moreover, with the proper selection of methods MSNs can be used for the delivery of hydrophilic and hydrophobic drugs.^28,29^ These nanocarriers have a large surface area of more than 500 m^2^ g^−1^, which allow them to behave as a dual-functional surface and making them appropriate for selective functionalization and modification of both internal and external surfaces.^30^ MSNs also have properties such as physicochemical stability, low mass density, excellent biocompatibility, biodegradability, controllable nanoparticle size and shape, and ease of large-scale synthesis and production, all of which make them promising and potential diagnostic and therapeutic candidates.^31–36^ MSNs have the advantages of tumor targeting, low toxicity, and controlled release of drug after their surface modification.^37–39^

United States Food and Drug Administration (FDA) has approved silica as “Generally Recognized as Safe”. In 2011, FDA approved silica nanoparticles in the form of Cornell dots (Cdots) for a first stage human clinical trial for targeted molecular imaging.^40^ In 2014, Philips *et al.* conducted a clinical trial for the first time on patients with metastatic melanoma using 124I-labeled Cdots for positron emission tomography (PET) imaging.^41^ In 2019, they also modified Cdots with different peptides such as cRGDY for molecular targeting of malignant melanoma cells.^42^ Recently (2022), peptide-modified Cdots were used for image-guided sentinel lymph node biopsy in head and neck melanoma in a nonrandomized clinical trial and the results showed that the use of peptide-modified Cdots is a safe and promising procedure to improve lymphatic mapping.^43^

Furthermore, the results of several *in vivo* biodistribution studies revealed that MSNs have a low toxicity and are mainly excreted through feces and urine after administration by various methods.^44,45^ Therefore, MSNs are excellent candidates as delivery systems for imaging and targeted therapies.^46,47^ In this review, we provided an overview of recent research advances and the perspective of future development of Cur-loaded MSNs in biomedical applications, with a particular focus on diagnostic, anticancer effects and bioimaging properties (Table 1).

**Table 1:** Summary of curcumin loaded-mesoporous silica nanoparticles synthesis and its biological activities^a^

No.	Scaffold	Type of study	Biological activities	Study samples and dosage	Finding	Ref.
1	5-Fluorouracil and Cur-loaded MSNs	*In vitro*	Anticancer effect (laryngeal cancer)	HepG-2 cells	↑ Apoptosis	48
					↑ Cell cycle arrest	
		*In vivo*		HepG-2 tumor-bearing nude mice (20 mg kg^−1^)	↑ Cleaved caspase 3	
					Encapsulation efficiency = 67.8%	
					Loading efficiency = 13.56%	
2	Cur-amine functionalized MSNs	*In vitro*	Curcumin bioavailability enhancement	Mice, oral administration (50 mg kg^−1^)	↑ Release profile	49
		*In vivo*			↑ Solubility	
					↑ Bioavailability	
3	Cur-loaded MSNs	*In vitro*	Cardioprotective effect	Doxorubicin-induced myocardial toxicity in rats (po, 200 mg kg^−1^)	↓ Malondialdehyde level	50
		*In vivo*		Oral gavage and pretreatment	↑ GSH, SOD and CAT in cardiac tissue	
					↑ Bioavailability	
4	pH-responsive	*In vitro*	Anticancer effect (glioblastoma cancer)	U87MG glioblastoma cancer cell line	↑ Solubility	51
	Cur-loaded chitosan MSNs				↑ Cur release with decreasing pH value of the media	
					IC_50_ = 5.21 μg mL^−1^	
					Encapsulation efficiency = 88.1%	
					Loading efficiency = 8.1%	
5	Redox-responsive	*In vitro*	Anticancer effect (breast cancer)	MDA-MB-231 breast cancer cells	↑ Precise targeting in tumors	52
	MSNs modified with hyaluronan (HA) or polyethyleneimine -folic acid (PEI-FA) *via* disulfide bonds	*In vivo*		Female BALB/c nude mice	↑ Accumulation in tumors	
				8 mg kg^−1^ every 3 d *via* the tail vein	↑ Biocompatibility	
					↓ Tumor growth	
6	Cur-amine functionalized MSNs	*In vitro*	Neuroprotective effect (α-synuclein fibrillation)	PC-12 cells	↑ Interaction with α-synuclein species	53
				α-Syn monomers	↓ Fibrillation of α-synuclein	
				100 μg mL^−1^	↓ Toxic effects of Cur	
					↑ Stability of Cur	
7	pH-responsive	*In vitro*	Anticancer effect (breast cancer)	Human breast cancer	↓ Cell proliferation	54
	Cur-loaded and calcium-doped dendritic MSNs modified with folic acid	*In vivo*		MCF-7 cells	↑ Intracellular ROS generation	
				BALB/c mice	↓ Mitochondrial membrane potential	
				30 mg kg^−1^	↑ Cell cycle retardation at G2/M phase and apoptosis rate	
					↑ Cur concentration in blood serum and tumor tissues	
					↓ PI3K/AKT/mTOR and Wnt/β-catenin signaling	
8	Plasmid and Cur-loaded MSNs	*In vitro*	Neuroprotective effect (neurodegenerative diseases)	N2a cells	↓ ROS-induced cell damage	55
				256 μg mL^−1^	↓ p-p38 expression	
					↑ Neurite outgrowth	
					↓ Nuclear translocation of NF-κB p65	
9	pH- and glutathione-responsive	*In vitro*	Cytotoxicity	MRC-5 human lung fibroblasts	↑ Cur release with decreasing pH	56
	Cur-loaded MSNs coated using tannic acid-Fe(iii) complex				↑ Cytotoxicity compared to free Cur	
					↑ Bioavailability and stability	
					↑ Cur release with increasing GSH concentration level	
					IC_50_ = 20.2 μM	
10	pH-responsive	*In vitro*	Anticancer effect	HeLa and NIH-3T3 cell lines	↓ Proliferation of HeLa and NIH-3T3 cell lines	57
	Chitosan-folate coated- amine functionalized MSNs				↑ Cytotoxic effect on cancerous HeLa cells	
					↑ Selective cellular uptake	
11	pH-responsive	*In vitro*	Anticancer effect (breast cancer)	MCF-7 (human breast carcinoma cell lines) A549 (human lung carcinoma cell lines)	↑ Anticancer activity	58
	Gold nanoparticles on the folate-conjugated dendritic MSNs				↑ Selective cellular uptake	
					↑ Photothermal potency	
					↑ Cur release with decreasing pH	
12	Cur-loaded PEGylated MSNs	*In vitro*	Anticancer effect (photodynamic therapy of cancer)	Cervical cancer cell line	↑ Endocytosis transport into cancer cells	59
				25 mg mL^−1^ to 0.39 mg mL^−1^	↑ Release of Cur	
					↑ ROS generation upon irradiation	
					↑ Bioavailability and solubility	
					Loading content = 8.1%	
					Loading efficiency = 89.1%	
13	pH-responsive Cur-loaded MSNs	*In vitro*	Anticancer effect (breast and colon cancer)	HCT-116 and MCF-7 cancer cells	↓ Cancer cells proliferation	60
				18, 14, 10, 6 μg	↑ Cur release with decreasing pH	
					↑ Stability of Cur	
					↑ Bioavailability and solubility	
					Loading efficiency = 80%	
14	pH-responsive	*In vitro*	Anticancer effect	OVCAR-5, CACO-2, CHLA, and MCF-7 cell lines	↑ Tumor specific toxicity	61
	Cur and naphthoquinone-loaded MSNs				↑ Cur release with decreasing pH	
					↑ Intensity, clear and distinct innate fluorescence that enabling detection and possible imaging applications	
15	Cur-loaded MSNs	*In vitro*	Anti-inflammatory effects	Male Wistar rats	↑ Anti-inflammatory activity comparable to diclofenac sodium	62
				Oral administration		
		*In vivo*		50 mg kg^−1^	↑ Biocompatibility	
					↓ Necrotic cells in the stomach compared to diclofenac sodium	
					↓ Necrotic cells in proximal tubules compared to diclofenac sodium	
16	Cur-loaded MSNs	*In vitro*	Hepatoprotective effect	CCl_4_-induced hepatotoxicity Wistar rats	↓ ALT and AST	63
		*In vivo*		Oral administration	↑ Necrotic hepatic cells in Cur-MSNs group than in the free Cur group	
				1.25 mL kg^−1^		
17	Cur-loaded chitosan functionalized-MSNs	*In vitro*	Anticancer effect (hepatocellular cancer)	Hep G2 cells	↑ Antioxidant activity	64
				100 μg mL^−1^	↑ Stability of Cur	
					↑ Cytotoxic activity	
					↑ LDH leakage	
					IC_50_ = 41 μg mL^−1^	
18	Self-fluorescent and stimuli-responsive	*In vitro*	Cytotoxicity	A549 cells	↑ Cur release with decreasing pH	65
	Cur-loaded MSNs			200 μg mL^−1^	↑ Bioavailability and stability	
					↑ Cur release with increasing GSH concentration level	
					↑ Fluorescent intensity	
					↑ Cytotoxic activity	
19	Asymmetric lollipop-like MSNs	*In vitro*	Antibacterial and anticancer activity (breast cancer)	MCF-7 cell	↓ Tumor cell proliferation	66
	Cur and gentamicin sulfate-loaded MSNs			*Escherichia coli*	↑ Tumor cell apoptosis	
				*Staphylococcus aureus*	↑ Cytotoxicity	
				2.5–13 μg mL^−1^	↑ Antibacterial activity	
					Loading capacity = 25.8 mg mL^−1^	
20	Cur-loaded hyaluronic acid Modified MSNs	*In vitro*	Anticancer effect (breast cancer)	MDA-MB-231 cells	↑ ROS generation	67
		*Ex vivo*		12 μg mL^−1^	↑ Cell cycle arrest at G2/M phase	
		*In vivo*		EAC-tumor bearing Swiss albino mice	↑ Cell apoptosis	
				10 mg kg^−1^	↓ NF-κB	
					↑ Bax, cleaved caspase 3	
					↓ Cell migration	
					↑ Antitumor efficiency of Cur	
					↑ Accumulation in the tumor tissue	
21	pH-responsive	*In vitro*	Anticancer effect (breast cancer)	MCF-7, 4T1, and MCF10A	↑ Cur delivery to breast cancer cells	68
	Cur-loaded guanidine functionalized PEGylated *I3ad* MSNs			10–60 μM	↑ Bioavailability	
					↑ Cur loading capacity	
					↑ Cytotoxic activity	
					↑ Cur release with decreasing pH	
					↑ Tumor cell apoptosis	
					IC_50_ for MCF-7 = 19.5 μM	
					IC_50_ for 4T1 = 14.68 μM	
					Loading content = 50%	
22	Cur and paclitaxel-loaded PEGylated lipid bilayer coated MSNs	*In vitro*	Anticancer effect (breast cancer)	Breast cancer cell lines	↓ Tumor burden	69
		*In vivo*		Female rats and mice intravenous and intratumoral administration	↑ Tumor site drug accumulation	
				2 mg kg^−1^	↓ Toxic side effects	
					↑ Cur delivery to breast cancer cells	
					↑ Cellular uptake	
23	Cur and paclitaxel-loaded PEGylated lipid bilayer coated MSNs	*In vitro*	Anticancer effect (breast cancer)	Canine breast cancer cells	↑ Sustained release effect	70
				1 μM	↑ Cytotoxic activity	
					Encapsulation efficiency = 30.7%	
24	Cur-loaded MSNs/nanofiber composites	*In vitro*	Supporting long-term proliferation and stemness preservation of human ADSCs	Human ADSCs	↑ Sustained release of Cur	71
					↓ Stem cell senescence	
					↓ p16^INK4A^	
					↑ Human telomerase reverse transcriptase	
					↑ Stemness potency in growing human ADSCs	
25	Cur-loaded β-Cyclodextrin functionalized PEGylated MSNs	*In vitro*	Anticancer effect (breast cancer)	MCF-7 and MCF10A	↓ Proliferation of MCF-7 cancer cell line	72
					↑ Cur release with decreasing pH	
					↑ Bioavailability and solubility	
					Loading efficiency = 88.55%	
26	Cur-amine functionalized MSNs	*In vitro*	Anticancer effect (bladder cancer)	HL-60 cell line	↑ Sustained release of Cur	73
			Nephroprotective effects	EJ cells	↓ Proliferation of cell lines	
				HEK-293 cells	IC_50_ for HL-60 = 2.8 μg mL	
					IC_50_ for EJ = 2.7 μg mL	
					IC_50_ for HEK-293 = 7.3 μg mL	
27	CUR-loaded MSNs and PLGA	*In vitro*	Anticancer effect (breast cancer)	MCF-7	↑ Cytotoxic activity	74
					↓ Cell migration	
					↑ Cell apoptosis	
					↑ Bax, caspase-3, and caspase-9	
					↓ Bcl-2 and hTERT	
					Encapsulation efficiency = 30.3%	
					Loading capacity = 9.25%	
					IC_50_ = 11.8 μg mL	
28	Stimuli-responsive	*In vitro*	Anticancer effect (lung cancer)	A549 cell	↑ Cytotoxic activity	75
	Triple-role Cur polymer shell-loaded MSNs			HeLa cells	↑ Cur release with decreasing pH	
					↑ Bioavailability and stability	
					↑ Cur release with increasing GSH concentration level	
29	Cur-loaded PEGylated MSNs	*In vitro*	Anticancer effect (liver cancer and cervical cancer)	HepG2	↑ Cytotoxic activity	76
		*In vivo*		HeLa	↑ Cellular uptake	
					↑ Cell cycle arrest at G2/M	
					↑ Biocompatibility and bioavailability	
					↑ Cur release with decreasing pH	
					Loading efficiency = 92.4%	
					IC_50_ for HepG2 = 20 μg mL	
					IC_50_ for HeLa = 28 μg mL	
30	pH-responsive	*In vitro*	Anticancer effect (breast cancer)	MCF-7	↑ Endocytosis transport into cancer cells	77
	Cur-loaded folate-receptor targeting MSNs			HEK-293T	↑ Cytotoxicity against MCF-7 cell than HEK-293T cells	
				10 μg mL^−1^	↑ Cellular uptake	
					↑ Cur release with decreasing pH	
					↑ Solubility of Cur	
					Loading efficiency = 9.5%	
31	pH-responsive	*In vitro*	*Salmonella* biosensor	*Salmonella* typhimurium	↑ Cur release with decreasing pH by addition of acetic acid	78
	Cur as signal reporter and ZnO-capped MSNs				Detection of *Salmonella* as low as 101 CFU mL^−1^ in 1.5 h	
					Loading efficiency = 40%	
					Loading content = 20%	
32	Temperature-responsive	*In vitro*	Anti-virus	A549 cells	↑ Antiviral activity	79
	Cur-loaded phosphorescence imaging metal ions (Eu^3+^ and Gd^3+^), PEGMA and MSNs			Zika virus	↑ Endocytosis transport into cells	
				156 μg mL	↑ Cur release at 39 °C	
33	Cur-loaded Polyethylenimine functionalized MSNs	*In vitro*	Anticancer effect (breast cancer)	MCF-7	↑ Cancer cell death	80
				MCF-7R	↑ Apoptosis of cancer cell by disrupting mitochondria and nucleus	
				20 μg mL^−1^	↓ Akt1	
					↑ Cleaved caspase 9	
					↓ IRE1α, PERK and GRP78	
34	Cur and siRNA-loaded MSNs	*In vitro*	Anti-inflammation	A549	↓ TNF-α release	81
				M0 macrophage-like cell line (dTHP-1)	↑ Anti-inflammatory effects	
				10 to 100 μg mL^−1^	Loading efficiency = 22%	
35	Cur-loaded zinc oxide nanoparticles decorated amine functionalized MSNs	*In vitro*	Wound healing	Sprague dawley rats (incision wound model)	↑ Wound healing rate	82
		*In vivo*	Antimicrobial activity	*S. aureus*	↑ Antimicrobial activity	
				*P. aeruginosa*	↑ Stability of Cur	
					↓ Wound width	
					Entrapment efficiency of Cur = 59%	
					Loading efficiency = 49.8%	

a ALT: alanine transaminase, AST: aspartate transaminase, GSH: glutathione, SOD: superoxide dismutase, CAT: catalase, NF-κB: nuclear factor kappa B, Bax: Bcl-2-associated X protein, Bcl-2: B-cell leukemia/lymphoma 2 protein, hTERT: telomerase reverse transcriptase, ROS: reactive oxygen species, PI3K: phosphoinositide 3-kinases, AKT: serine/threonine protein kinase, mTOR: mammalian target of rapamycin.

## Synthesis of Cur loaded-MSNs

### Amine-functionalized MSNs

**Fig. 2:**
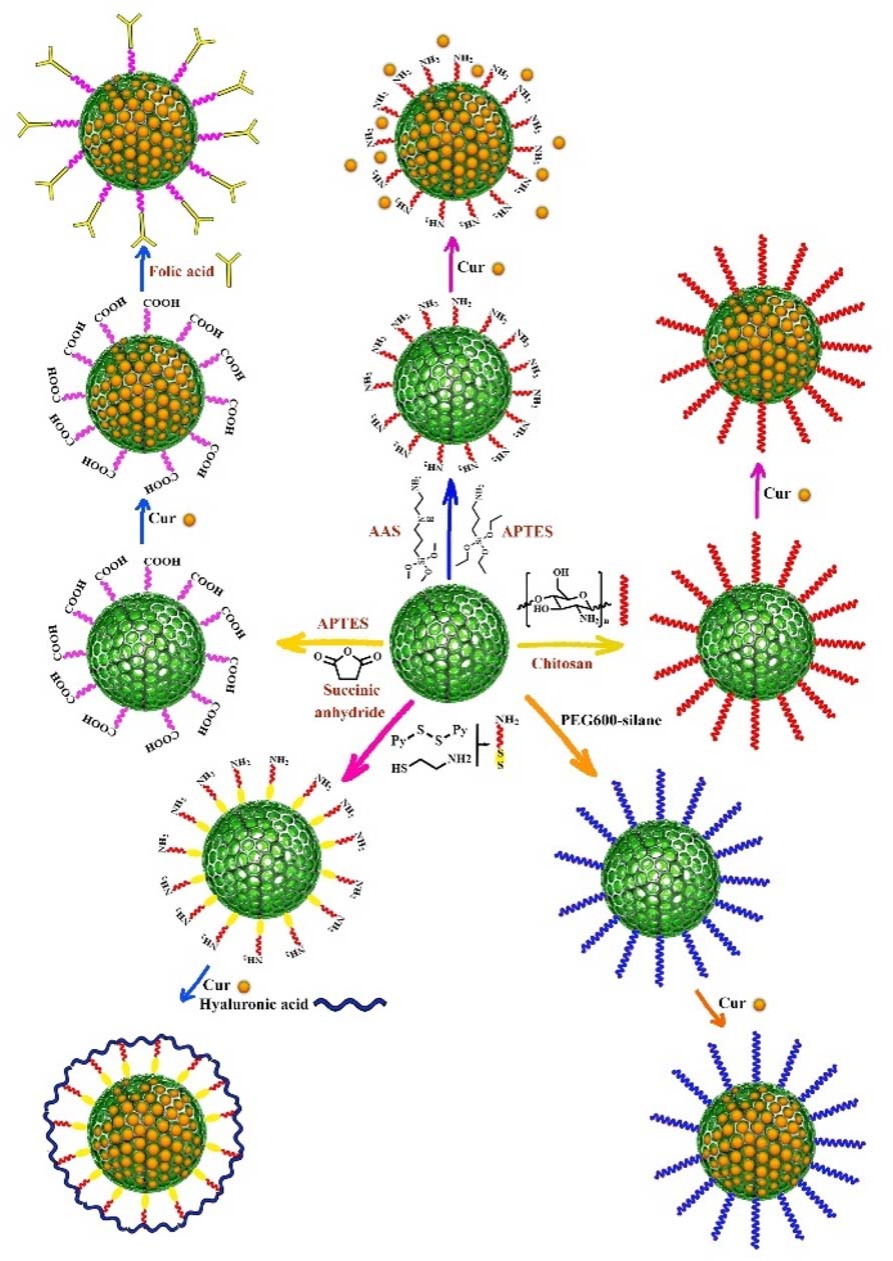
Different synthesis methods of curcumin loaded-mesoporous silica nanoparticles.

Surface functionality is a key factor for drug carriers, and MSNs have the ability to be functionalized with various organic groups, making them potential candidates for interaction between a wide range of drugs/molecules and functionalized surfaces.^83^ Amine functionalized-silica nanoparticles have been demonstrated to be an effective approach for providing a controllable release profile for a variety of molecules.^84,85^ As it has been previously reported in the literature, hydrogen bonding between the phenolic group of Cur and the primary amine group increased Cur binding and stability.^86^ Furthermore, the spectral data revealed weak interaction of Cur complexes with the surface of amino-functionalized MSNs *via* NH^3+^–O^−^ interactions.^73^ In several studies, 3-aminopropyltriethoxysilane (APTES) was employed as an amine functionalization agent on the surface of MSNs, and Cur was then loaded onto the amine functionalized-MSNs.^62,63^ Hartono *et al.* synthesized cubic MSNs (particle size and pore size around 100 and 10 nm, respectively) and mesoporous silica micron-sized-particles (MSM) that were functionalized with 3-aminopropyltriethoxysilane (APTES) and then Cur was encapsulated within amine functionalized-MSN and MSM (Fig. 2). The *in vitro* release and solubility study of Cur indicated that Cur loaded-amine functionalized MSN had higher solubility and better release profile compared to Cur loaded-amine functionalized MSM. Both Cur loaded-amin functionalized MSN and MSM showed significantly higher bioavailability than free Cur. The maximum Cur concentration (*C*_max_) was estimated 0.0291 μg mL^−1^ in mice plasma.^49^

KIT-6 silica with a cubic Ia3d mesostructure and KIL-2 silica with a textural mesoporosity were also employed as nanocarriers for Cur loading, both of them were functionalized using APTES. KIT-6 had a greater amino group content (8.5 wt%) than KIL-2 (5.7 wt%).

One of the main factors that influences the content of Cur loading and the rate of Cur release are its loading methods into the MSNs. Overall, Cur loaded-MSNs by solid-state method show a slower drug release profile when compared to the samples prepared by incipient wetness impregnation loading method. It can be explained by the lower adsorbed amount of Cur in KIL-2 prepared by incipient wetness impregnation compared to solid state method. Furthermore, in wet impregnation method, the drug encountered in the formulations has more soluble amorphous state, while in the solid-state analogs, there is less water-soluble crystalline form of Cur.^73^ Cur loaded amine-functionalized KIL-2 demonstrated faster Cur release due to its more open pore structure with much higher pore sizes as compared to that prepared with the KIT-6. More ordered 3D pore structure of KIT-6 with interconnected channels leads to the trapping of Cur within the pores and its slower release rate.^73^

The functionalization of MSNs with functional molecules containing more than one amine group significantly increases the amount of loading capacity and efficiency of Cur onto MSNs. For example, APTES and 3-(2-aminoethyl amino) propyltrimethoxysilane (AAS) contain one and two amine groups, respectively, and AAS-functionalized MSNs had significantly higher loading capacity of Cur (33.5%)^53^ than APTES-functionalized MSNs (3.6%) (Fig. 2).^87^ Cur loaded onto AAS-MSNs exhibited considerably lower cytotoxicity than free Cur. The electrostatic and hydrophobic interactions between functional groups on the surface of AAS-MSNPs and Cur molecules resulted in higher loading capacity and efficiency for Cur. Furthermore, the stability of Cur increased compared to free Cur.^53^

Pote *et al.* recently synthesized APTES-functionalized MSNs that were subsequently decorated with ZnO nanoparticles. Cur was then loaded onto ZnO-MSNs using the incipient wetness impregnation process and employed as a tissue adhesive for wound healing applications. The drug encapsulation and drug loading efficiency were calculated to be 59% and 49.80%, respectively.^82^ Magnetic MSNs are also used to load Cur as an efficient carrier in drug delivery systems.^88^ Saputra *et al.* used magnetite nanoparticles to improve hard-templating method and increase the storage capacity of MSNs and therefore load more Cur within their mesoporous structure.^89^

Recently, Atiyah *et al.* synthesized amine-functionalized silica nanoparticles for Cur delivery using SBA-15 and MCM-41. CTAB and P123 were used as surfactant for MCM-41 (ref. 90) and SBA-15,^91^ respectively. The best drug loading efficiency for amine-functionalized MCM-41 and amine-functionalized SBA-15 was 80 and 89.7%, respectively. The release profile of amine-functionalized MCM-41 and amine-functionalized SBA-15 showed maximum release of 29.95% and 41.2% for Cur, respectively.

Alginate oligosaccharide is a biodegradable acidic polymer with low toxicity and high solubility. Liu *et al.* synthesized amine-functionalized MSNs coated by alginate oligosaccharide to obtain the pH-responsive release of Cur and improve its bioavailability. The amino modification on the surface of MSNs performed by APTES, and then Cur was loaded into the amine-functionalized MSNs. After that, the obtained nanoparticles were coated with alginate oligosaccharide in the presence of 1-(3-dimethylaminopropyl)-3-ethylcarbodiimide and *N*-hydroxysuccinimide to activate carboxyl groups and form amide bonds. The total release rate of Cur from alginate oligosaccharide-encapsulated MSNs at neutral and acidic pH was 28.9% and 67.5%, respectively. The results of *in vitro* experiment indicated that alginate-coated MSNs are easily absorbed by colon cancer cells compared to free Cur and also have significantly higher cytotoxicity than free Cur.^92^

Otri *et al.* loaded MSNs with rhodamine B and then decorated with APTES and finally capped with Cur to prepare a selective and sensitive structure for the fluorogenic detection of human serum albumin with a detection limit of 0.1 mg mL^−1^ at neutral pH. Rhodamine B is selectively released from nanoparticles in the presence of albumin due to the opening of pores (uncapping of the nanoparticles) caused by the formation of a complex between albumin and Cur.^93^

### Chitosan-functionalized MSNs

Chitosan is a natural biodegradable linear polysaccharide composed of a large number of primary amine groups that acts as a pH-sensitive polymer. It swells in acidic media of cancerous tissues and this feature allows the polymer to differentiate between normal and cancerous cells and provides controlled drug release.^94–98^

Cur is loaded into chitosan-MSNs nanocarriers through a simple adsorption method. First, chitosan-capped MCM-41 nanoparticles are synthesized by covalent coupling of chitosan amino groups and (3-glycidyloxypropyl) trimethoxysilane on the surface of MCM-41. Then, different amounts of Cur are added to the chitosan-MSNs suspension and allowed to adsorb onto the surface of the nanoparticles under gentle stirring for several hours. The loaded Cur is then entrapped within the chitosan-MSNs nanocarriers through the formation of hydrogen bonds and electrostatic interactions between Cur and the chitosan and MCM-41 surfaces.

Chitosan-capped MCM-41 were used as a pH-responsive nanocarrier for Cur delivery and prepared through the reaction of chitosan amino groups and (3-glycidyloxypropyl) trimethoxysilane as a covalent coupling agent which resulted in the formation of a secondary amine on the surface of MCM-41. Subsequently, different amounts of Cur were loaded into chitosan-MSNs nanocarriers (Fig. 2).^51^ This pH-responsive nanocarrier increased solubility and completely dispersed in aqueous media without any aggregation. Furthermore, chitosan promoted the anticancer activity of Cur against glioblastoma cancer cell line. Based on the findings of this study, Cur and Cur-loaded chitosan-MCM-41 had IC_50_ values of 15.20 and 5.21 μg mL^−1^, respectively, after 72 hours of incubation against glioblastoma cancer cell line. Moreover, this nanocarrier showed a sustained and slow release of Cur in 96 hours at acidic pH compared to the pH of the environment.^51^

Chitosan's terminal amino groups involved in the synthesis of biomimetic silica nanoparticles by catalyzing the hydrolysis/condensation of the silica source and subsequent silica aggregation.^99^ The aim of biomimetic synthesis method is the synthesis of target molecule through a series of reactions. Kong *et al.* synthesized silica co-encapsulated Cur nanoparticles by biosilicification method with the average particle size of 75 nm. They found that coating Cur loaded-MSNs with chitosan increased the stability of Cur and its cytotoxic effects against hepatocellular carcinoma cells compared to free Cur and Cur loaded-MSN. Cur loaded chitosan-functionalized MSNs significantly increased LDH leakage with longer duration compared to Cur and Cur-loaded MSN.^64^

### Folic acid-functionalized MSNs

The conjugation of MSNs with folic acid, which has a high affinity for the receptors overexpressed on the surface of cancer cells, increases MSN uptake in targeted cells.^100–102^ In order to load Cur onto the surface of MSNs for breast cancer treatment, Wang *et al.*^54^ employed dendritic MSNs with center-radial pore channels and open 3D dendritic superstructures. The surface of MSNs was modified with folic acid, and the nano pores of folic acid-functionalized MSNs were immobilized with calcium hydroxide to provide pH-responsive release, and Cur was loaded by chelating with divalent calcium. This Cur delivery system improved Cur intracellular release and distribution, while also increased anticancer activity against MCF-7 breast cancer cells, which can be attributed to targeted delivery *via* folic acid-functionalized MSNs, pH-responsive release profile, and high biocompatibility.^54^

The strong electrostatic interaction and pH-sensitive Schiff base reaction between the carbonyl group of Cur and the amine group of APTES-functionalized MSNs allows Cur to be loaded into the APTES-functionalized MSNs. Chen *et al.*^77^ used APTES-functionalized MSNs that were conjugated with folic acid and loaded with Cur *via* a pH-sensitive Schiff base reaction (folic acid–MSN–N=C–Cur). This nano drug delivery system had a high amount of drug loading capacity (9.5 ± 0.2%) and could effectively target folic acid-receptor-rich MCF-7 cells through endocytosis mediated by folic acid receptors (Fig. 2).

In another study, MSNs were functionalized with folic acid-conjugated chitosan which acts simultaneously as a double pH-responsive and active targeting drug delivery system. For this purpose, APTES-functionalized MSNs with normal and large pore sizes were conjugated with succinic anhydride and then Cur was loaded onto the synthesized MSNs to develop this drug delivery system. Finally, the surface of carboxylic acid-functionalized MSNs was electrostatically coated with a combination of folic acid and chitosan. The findings revealed that the large pore size MSNs had a 2-fold higher loading efficiency and capacity for Cur than normal pore size MSNs and Cur was released *via* Korsmeyer–Peppas mechanism.^57^ The pore size of normal MSNs and large MSNs was 24.18 and 36.38 Å respectively. The chitosan-folic acid coating layer increased the overall particle size (167 nm) compared to large pore size MSNs without a coating layer (72.2 nm).^57^

Thiol-functionalized MSNs have been also used as multifunctional MSNs for targeted drug delivery *via* redox-responsive system. Li *et al.*^52^ synthesized redox-responsive systems by modifying thiol-functionalized MSNs with hyaluronan or polyethyleneimine-folic acid *via* disulfide bonds. 2,2-Dipyridyl disulfide and 3-mercaptopropionic acid were used to prepare disulfide bonds and the surface of MSNs was functionalized with amino- and carboxyl group, respectively (Fig. 2). Hyaluronan and polyethyleneimine-folic acid were then conjugated to the surface of MSNs *via* amidation. Both ligands act as gatekeepers that preserve the Cur loaded in the pores and allow it to be selectively released into tumor cells. Redox-responsive disulfide bonds breakdown in the presence of reducing thiols such as glutathione (GSH), which present with higher concentrations in cancerous cells than in normal cells.^103,104^ According to the findings, folic acid-functionalized MSNs had a higher cellular uptake, more specific targeting ability and greater accumulation in tumors than that of hyaluronan-functionalized MSNs.^52^ Hyaluronic acid-modified MSNs were also synthesized for targeted delivery of Cur in breast cancer cells through the reaction of APTES-functionalized MSNs with hyaluronic acid followed by Cur loading in the next step.^67^

Dendritic MSNs coated reduced graphene oxide nanosheets were also employed as sandwich-like nanocomposite and multifunctional MSNs for Cur targeted delivery and cell imaging. The surface of nanocomposite was further functionalized *via* 3-(trimethoxysilylpropyl)diethylenetriamine to obtain an amine-functionalized MSNs. Folic acid was conjugated onto the nanocomposite *via* amide bond formation. Cur was loaded into the mesoporous nanocomposite (loading capacity = 18.34%) *via* its adsorption in the channels of MSNs and its π–π interaction with reduced graphene oxide. The findings revealed that low pH and near-infrared (NIR) laser irradiation reduce the hydrophobic interaction between Cur and graphene, and also decrease the electrostatic interaction between Cur and MSNs, thus it can stimulate the release of Cur under the thermal and pH-responsive conditions. Furthermore, immobilization of gold nanoparticles onto the surface of this nanocomposite enabled it to act as a promising photothermal therapeutic agent for many cancers.^58^

### PEGylated MSNs

FDA has approved poly ethylene glycol (PEG) as a safe, water-soluble substance providing a long and safe circulation of drugs. PEGylated MSNs have been employed as nanocarrier for Cur delivery. For the synthesis of this nanocarrier, PEGylation of MSNs can be done before or after Cur loading. As an example, Elbialy *et al.* loaded Cur into the MSNs matrix by electrostatic interactions, after which it was PEGylated using PEG_4000_. Cur release profile was pH-responsive, with a high release rate in acidic medium which is due to the dissociation of the bonds between Cur and MSNs, as well as the collapse of PEG at acidic pH.^76^ PEGylation of MSNs increases Cur biocompatibility, stability and permeability, enhances the circulation time of the nanodrug delivery system in the blood, and prevents phagocytosis through the reticuloendothelial system.^76,105^

Recently, poly(ethylene glycol) mono-methyl ether was used to functionalize MSNs and modified with succinic anhydride to prepare PEG-COOH. APTES-modified MSNs were functionalized with PEG-COOH *via* amidation. Cur was then loaded into the pores of PEGylated MSNs through hydrophobic interactions using the centrifugal immersion technique and the drug loading content and drug loading efficiency were reported to be 8.1% and 89.1%, respectively.^59,106^

In two studies, PEGylated KIT-6 was used as the nanocarrier and prepared *via* the reaction between PEG_600_–silane and KIT-6 silica. PEG_600_–silane was synthesized *via* the nucleophilic addition of the end hydroxyl group of PEG_600_ to the isocyanate group of 3-(triethoxysilyl) propyl isocyanate (TESPIC) (Fig. 2). In one of these studies, PEGylated KIT-6 was functionalized with guanidine and then Cur was loaded into the surface of nanocarrier pores. In fact, guanidine functionalized-PEGylated KIT-6 demonstrated a greater loading capacity of Cur into the MSNs pores due to the electrostatic interaction between guanidine functional groups and carbonyl group of Cur.^68^ In another study, PEGylated KIT-6 was functionalized with β-cyclodextrin *via* the nucleophilic attack of PEG hydroxyl group to the tosylated β-cyclodextrin. Cur was then loaded into the surface of nanocarrier pores and formed the complex with β-cyclodextrin.^72^

Lin *et al.* used PEGylated lipid bilayer to coat MSNs in order to prevent uncontrolled and premature release of loaded Cur and paclitaxel from the surface of MSNs. For this purpose, they loaded paclitaxel and Cur into the pores of MSNs. PEGylated lipid bilayer was then wrapped around the MSNs. They found that the thickness of the lipid bilayer is an important factor that affects the function of PEGylated lipid bilayer coated MSNs, and the appropriate thickness prevents premature or delayed drug release.^69,70^

Recently, Ho *et al.* designed and synthesized silica-containing redox nanoparticles using amphiphilic redox polymers such as poly(ethylene glycol)-b-poly[4-(2,2,6,6-tetramethylpiperidine-*N*-oxyl)aminomethylstyrene] (PEG-b-PMNT) to improve gastrointestinal distribution of Cur *via* oral administration and enhance its antioxidant activity. The cytotoxicity of silica-containing redox nanoparticles was much lower than free Cur against normal fibroblasts L929 cell line. The results of the *in vivo* experiment showed a higher accumulation of Cur in the intestine and colon of mice than those treated with free Cur, that is due to the accumulation on the mucus layer by the PEG tail in the nanoparticle structure. It can be considered as a promising drug delivery system for the treatment of inflammatory bowel disease.^107^

### Cur-loaded MSNs/nanofiber composites

Electrospinning technique was recently employed to encapsulate Cur-loaded MSNs into a nanofibrous mat, resulting in the formation of an electrospun nanofiber-mediated drug release platform. While anticancer drugs kill cancer cells in nanofiber-mediated drug delivery systems, ultra-fine nanofibers promote tissue reconstruction in tumor defects by providing an adequate microenvironment.^108^ Cur can be loaded into amine-functionalized MSNs and subsequently encapsulated into polymers such as poly(lactic-*co*-glycolic acid) (PLGA) nanofibers^74^ and poly-ε-caprolactone/gelatin (PCL/GEL)^71,109,110^ hybrid nanofibers using electrospinning method to produce nanofiber-mediated Cur delivery systems.

The co-encapsulation of Cur loaded MSNs with a hybrid of PCL/GEL nanofiber effectively enhanced the antitumor activity against MDA-MB-231 breast cancer cells, that could serve as locally implantable scaffolds for potential postoperative breast cancer treatment.^109^ This scaffold was also used for the long-term cultured human adipose-derived stem cells for effective stem cell-based regenerative therapies. According to the dual stage release (initial fast release and late extended drug release) of Cur from MSNs/electrospun nanofibers, human adipose-derived stem cells cultured on this medium demonstrated the highest adhesion, metabolic activity and proliferation rate with fibroblastic phenotype after 28 days of culture. Furthermore, this scaffold significantly increased the telomerase activity and telomere length compared to control group.^110^

Liu *et al.*^75^ synthesized bis-acrylated Cur that was then loaded onto MSNs through the thiol–ene coupling reaction. The reaction of bis-acrylated Cur with 3-mercaptopropyltrimethoxysilane functionalized-MSNs resulted in the formation of β-thioesters, which can be hydrolyzed in the presence of GSH. The results of the fluorescent property analysis revealed that Cur polymer has more significant fluorescence intensity even in aqueous PBS dispersion, which makes it suitable for cell fluorescence imaging.^75^

### Cur-loaded liposome based-MSNs

**Fig. 3:**
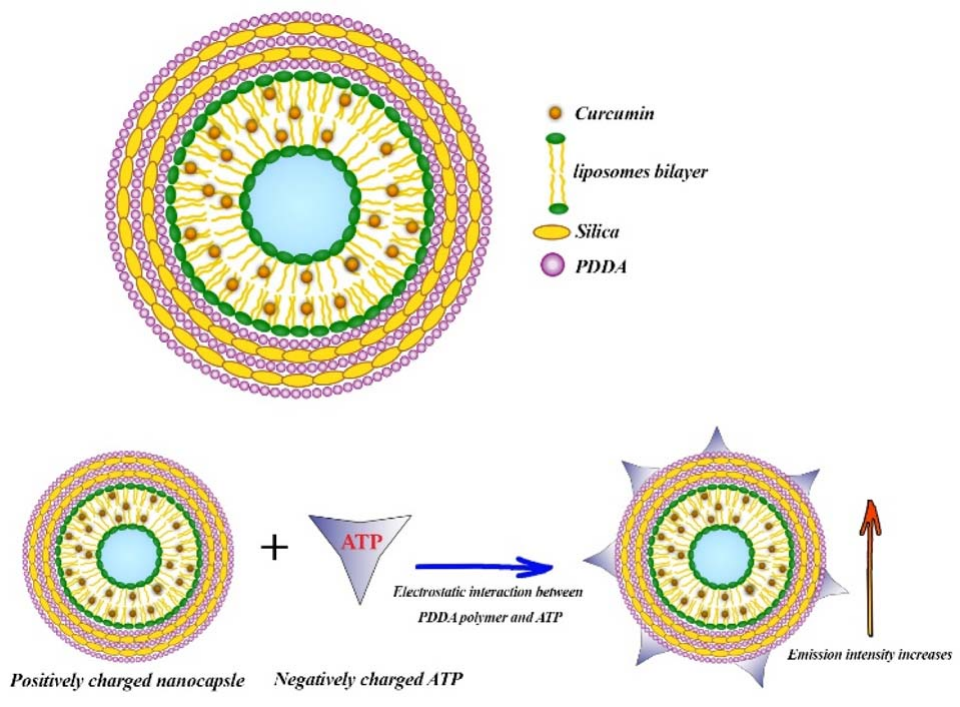
Adenosine triphosphate (ATP) estimation by DMPC nanocapsules.

Phospholipids such as 1,2-dimyristoyl-*sn*-glycero-3-phosphocholine (DMPC) form liposomes after being dispersed in water, which can be used as effective drug delivery vehicles due to the encapsulation of hydrophobic molecules within the lipids bilayers and the hydrophilic molecules within the liquid core.^111^ Patra *et al.* designed and synthesized poly (dimethyldiallylammonium chloride) (PDDA) and SNs-coated DMPC liposomes in order to enhance the fluorescence intensity of Cur. They prepared liposomal Cur by thin film hydration method and subsequently pH 7 buffer solution was added to the thin film and vigorously vortexed to obtain a completely homogeneous solution. For nanocapsule synthesis, liposomal Cur was first coated by PDDA as a cationic polyelectrolyte. In the next step, silica nanoparticles were added and then coated again with PDDA. After that, the second layer of silica nanoparticles was added and the final nanocapsule was coated with a PDDA layer (Fig. 3). The 3-layer PDDA acts as stabilizer to coat both the negatively charged DMPC liposomes and silica nanoparticle. PDDA layers also prevent the rapid leakage of Cur. This DMPC nanocapsule was used to detect adenosine triphosphate (ATP), which was determined through changes in the fluorescence emission of the nanocapsule. With increasing ATP concentration, the fluorescence intensity of nanocapsules increased while no response of free Cur to ATP was observed. ATP binds to the polymer through electrostatic interaction, and on the other hand, the excited state of Cur is stabilized in the hydrophobic lipid bilayer structure (Fig. 3).

The limit of detection of ATP was 0.5 μM.^112^ Recently, in another study, Patra *et al.* synthesized three different nanocapsules as follows: liposomal Cur coated with a layer of PDDA, liposomal Cur coated with PDDA–silica–PDDA layer and the last nanocapsule consists of liposomal Cur–PDDA–silica–Cur–PDDA layer. They found that the fluorescence intensity of Cur in these nanocapsules considerably increased by 25, 54 and 62-fold, respectively. By increasing the number of layers on the surface of liposomes, the release of Cur was significantly decreased. The results of cytotoxicity assay showed that the anticancer effect of the nanocapsule consist of liposomal Cur–PDDA–silica–Cur–PDDA layer on MCF-7 cells was significantly higher than the other two nanocapsules and the free serum.^113^

## Environment-responsive therapy using Cur-loaded MSNs

Cur may leak from mesopores of MSNs during blood circulation and penetration of Cur-loaded MSNs into tumor tissue, resulting in an insufficient Cur uptake at the tumor site. Active targeting therapy using Cur-loaded MSNs results in higher efficacy and accumulation of Cur in targeted cells or tumors. Hence, most studies used modified MSNs with environment-responsive gatekeepers. Tumor tissue microenvironment has a more acidic pH with a range of 4.5–6.5, higher glutathione concentration (2–10 mmol L^−1^) and higher temperature (40–42 °C) than normal tissue. Therefore, Cur-loaded MSNs can be modified by pH-responsive, redox-responsive, and temperature-responsive gatekeepers for active targeting therapy.

### pH-responsive Cur-loaded MSNs

**Fig. 4:**
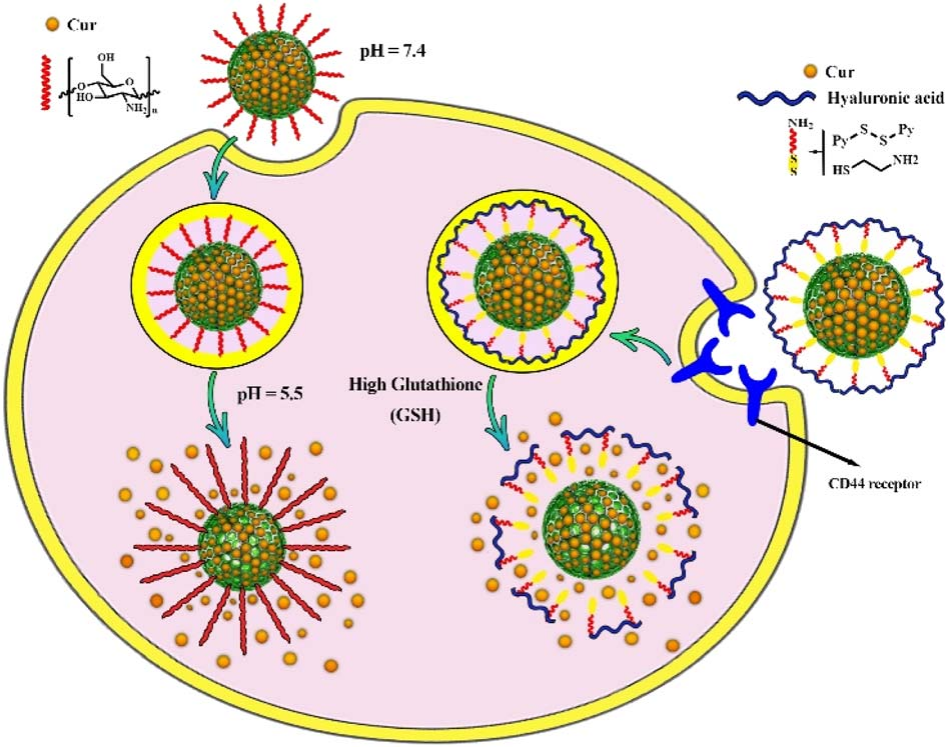
Controlled drug delivery from pH-responsive curcumin-loaded MSNs and glutathione (GSH)-responsive curcumin-loaded MSNs and their release mechanisms.

Elbialy *et al.* investigated the release profile of Cur-loaded MSNs and found that under normal physiological conditions (pH 7.4) only less than 20% of Cur was released during 13 days. With decreasing pH value of the medium (pH ∼ 5.5) approximately 94% of the encapsulated Cur was released in the medium during 13 days, indicating pH-dependent solubility behavior of Cur and its potential for high sustained release in the tumor microenvironment, which is considered as an effective way for targeted drug delivery.^60^ Some functionalized MSNs can act effectively as a pH-responsive nanocarrier and improve these properties for more targeted Cur delivery.^114^ As an example, chitosan-functionalized MSNs were used as pH-responsive nanocarrier. The *in vitro* release kinetic study of Cur for chitosan-functionalized MSNs revealed that the rate of Cur release from nanocarrier increased with decreasing pH value of the media (from pH 7.4 to pH 5.5). In acidic media, the amino groups of chitosan on the surface of the nanocarrier are protonated, converting chitosan to a cationic polyelectrolyte. This leads to repulsion in the chitosan polymer chains and allows it to swell. As the pH raised, the deprotonation of amino groups increased while repulsion in polymer chains and water absorption decreased, allowing chitosan polymeric chains to shrink and form layers surrounding MSNs. As a result of coating the porous surface of MSNs with layers of chitosan polymer chains, the loaded Cur cannot be released (Fig. 4).^51,57,115^

Calcium-doped dendritic MSNs act as pH-responsive nanocarrier. Cur was loaded on the surface of calcium-doped dendritic MSNs by chelating with divalent calcium. According to the findings of the drug release study, Cur release from calcium-doped dendritic MSNs modified with folic acid in neutral PBS solution was approximately 35% after 12 hours, while the release rate considerably increased in acidic PBS solution (pH 5.0) to 80% after only 0.5 hours. Under acidic conditions, Cur and Ca^2+^ complex on the surface of MSNs can be dissociated, resulting in faster release of Cur and increasing its solubility. Calcium-doped dendritic MSNs were well dispersed in aqueous solutions and showed color changes from dark red color in aqueous media to light yellow when the pH decreased to 5.0.^54^

Graphene oxide nanosheets coated by dendritic MSNs were used as pH-responsive nanodrug delivery system and Cur was loaded in the channels of MSNs through the π–π interaction with reduced graphene oxide. According to the results of an *in vitro* drug release study, the maximum cumulative Cur release was observed in stomach gastric juice pH (pH = 2.7) and tumor tissue microenvironment pH (pH = 5.7), which can be related to a decrease in the hydrophobic interaction between Cur and graphene as well as a reduction in the electrostatic interaction between Cur and MSNs.^58^

Jung *et al.* developed a pH-responsive nanocarrier based on host–guest interactions. In this study, the surface of MSNs was functionalized with phenanthroline by formation of covalent bonds. After that Cur was loaded into pores of functionalized MSNs and these pores were closed by the formation of coordination bonds between the phenanthroline group and Cu^2+^ as a host–guest carrier. In acidic media (pH ∼5), Cu^2+^ can be easily exchanged with proton and the nitrogen atoms of phenanthroline groups become protonated. Therefore, by dissociating the host–guest complex, Cur can be released from the pores of MSNs into the solution phase.^116^

Cur-naphthoquinone conjugate has recently been exploited as a novel theranostic molecule loaded into MSN pores that has the potential to be used for cancer targeting, diagnosis and therapy.^14,61^ Cur-naphthoquinone-loaded MSN effectively reduced the cell viability of various cell lines including OVCAR-5, CACO-2, CHLA, and MCF-7 to below 50%. It also showed tumor-specific toxicity and therefore significantly reduced cell viability in cancer cell lines compared to a healthy fibroblast cell line. Microscopic images demonstrated the detection capabilities of naphthoquinone-loaded MSNs for molecular imaging purposes due to their high-intensity intrinsic fluorescence.^61^ The release profiles (pH = 7.4 and 6.8) of Cur-loaded MSNs and Cur-naphthoquinone-loaded MSNs were statistically significant. Cur-loaded MSNs showed no pH specificity at pH values of 7.4 and 6.8 (*p* = 0.31), whereas Cur-naphthoquinone-loaded MSNs exhibited robust pH-responsivity. At acidic pH, naphthoquinone undergoes a reduction reaction and forms hydronaphthoquinone which has a higher solubility than the naphthoquinone form. This reaction is strongly dependent on pH, thus allowing Cur-naphthoquinone-loaded MSNs to exhibit a great pH-responsive release profile.^61^

Likewise, guanidine-functionalized MSNs showed a pH-responsive profile for Cur release. According to the release curves, about 100% of the loaded Cur was released at pH = 5.4 after 120 hours, whereas 52.41% was released at pH = 7.4. The guanidine functional groups and the carbonyl group of Cur interact electrostatically, in which these hydrogen bonds dissociate at acidic pH and release Cur into the medium.^68^

Elbialy *et al.* developed Cur-loaded-PEGylated MSNs that demonstrated a pH-responsive release profile of Cur, with a high release rate in acidic medium due to the dissociation of electrostatic interactions between Cur and MSNs, as well as the collapse of PEG at acidic pH.^76,117^

### Glutathione (GSH)-responsive Cur-loaded MSNs

In several studies, tannic acid has been employed as a non-surfactant template for the synthesis of MSNs. Tannic acid has the ability to form a complex with polyvalent cations like Cd^2+^, Co^2+^, Cr^3+^, Zn^2+^ and Fe^2+^.^118–121^ Kim *et al.* developed tannic acid-Fe(iii) complex deposition on the surface of MSNs to provide the pH- and glutathione-responsive nanocarrier. The results of *in vitro* release study revealed sustainable Cur release at pH 7.4, while a rapid Cur release was observed by lowering the pH to 6.0 or 4.5. Furthermore, GSH-responsive release of Cur indicated that the carboxylate and amine groups of GSH can accelerate the decomposition of tannic acid-Fe(iii) complex *via* competitive complexation with iron. According to the cytotoxicity assay, the IC_50_ values of free Cur, MSN and tannic acid-Fe(iii) complex MSN were >150 μM, while the IC_50_ value for Cur loaded-tannic acid-Fe MSN was 20.2 μM for the treatment of MRC5 cells.^56^

Thiol-functionalized MSNs can act as stimuli-responsive nano drug delivery system. The thiol–ene coupling between bis-acrylated Cur and 3-mercaptopropyltrimethoxysilane functionalized-MSNs can form β-thioesters and Cur polymer shell, which can be hydrolyzed in the presence of GSH. The hydrolysis of β-thioesters would result in the degradation of the Cur polymer shell, which would then open the pores of MSNs and allow the Cur and drugs (doxorubicin) encapsulated in the pores to be released. The quantity of Cur polymer shell and GSH concentration affect the hydrolysis rate.^65,75^ Liu *et al.* designed stimuli-responsive MSNs using large pore MSNs in which Cur gatekeeper was anchored to the surface of MSNs through thiol–ene “click” chemistry and then the surface of the nanocarrier was coated by F127 through hydrophobic interactions and self-assembly. The results of MTT assay showed that the cell viability of A549 cells when treated with nanocarrier treatment for 48 hours was 100%, while the treatment with Cr loaded nanocarrier showed less than 48% cell viability at the concentration of 200 μg mL^−1^, indicating the good biocompatibility of nanocarrier.^65^

Modifying thiol-functionalized MSNs with hyaluronan or polyethyleneimine-folic acid *via* disulfide bonds resulted in the formation of redox-responsive drug delivery system. Both ligands act as gatekeepers that preserve the Cur loaded in the pores and allow it to be selectively released in the presence of GSH (Fig. 4). The *in vitro* drug release study revealed that the breakdown of disulfide bonds in the presence of GSH (10 mM), resulted in the release of almost the entire Cur (96%) from both modified MSNs with hyaluronan or polyethyleneimine-folic acid, as compared to the release of both modified MSNs (48% and 49% release, respectively) in the absence of GSH.^52^

Recently, fucoidan has been used as a sulfated polysaccharide to form disulfide bonds on the surface of MSNs, which is considered as a glutathione-responsive nanocarrier for Cur delivery in the tumor microenvironment.^122^

### Temperature-responsive Cur-loaded MSNs

Photothermal therapy using near-infrared (NIR) laser photo absorbers can be used in combination with nano drug delivery system to achieve their optimal therapeutic efficacy. In a recent study, reduced graphene oxide nanosheets were coated with dendritic MSNs, which were subsequently functionalized with an amine group and coupled with folic acid. After that, the folate-functionalized nanocomposite was immobilized with Au nanoparticles and loaded with Cur. The *in vitro* drug release study indicated that the release rate of Cur is higher in the presence of NIR laser as an external heat-stimulator. The laser irradiation accelerates the dissociation of Cur from the folate-functionalized nanocomposite. At pH = 5.7 (tumoral microenvironment pH), the cumulative release of Cur after 60 minutes of NIR laser irradiation was approximately three times more than without NIR laser irradiation. The findings revealed that a low pH and NIR laser irradiation (temperature rise) decrease the hydrophobic interaction between Cur and graphene, as well as decrease in the electrostatic interaction between Cur and MSNs, thus it can stimulate the release of Cur under the thermal and pH-responsive conditions.^58^

Lo *et al.* developed a temperature-responsive nanocarrier using poly(ethylene glycol) methacrylate (PEGMA) as a temperature-responding polymer as well as Eu^3+^ and Gd^3+^ as magnetic and fluorescence imaging metal ions. After that, the surface of MSNs modified with PEGMA and loaded with Cur. The results of *in vitro* release investigation revealed that the amount of released Cur increased from less than 20% to more than 70% when the temperature was raised from 34 °C to 38 °C. It is due to changes in the brush-shaped arrangement of PEGMA, which can be opened and released the encapsulate Cur by increasing the temperature. This multifunctional nanocarrier was used as antiviral agent against Zika virus and MSNs encapsulated Cur showed more antiviral activity than free Cur.^79^

## Co-delivery of anticancer drug with Cur-loaded MSNs

Recently, the anticancer effects of co-delivery of 5-fluorouracil with Cur-loaded MSNs were investigated on Hep-2 laryngeal cancer cells as well as Hep-2 tumor-bearing nude mice. According to the findings, MSNs could deliver 5-fluorouracil and Cur to tumors in mice and synergistically promoted cell cycle arrest and apoptosis in Hep-2 cells. Furthermore, when MSNs were utilized as a nanocarrier, the synergistic effects of 5-fluorouracil and Cur were enhanced and the drug dose necessary for effectiveness was reduced compared to when both drugs were administered alone. MSNs also displayed no cytotoxicity or adverse effects in mice, and had no cytotoxicity in Hep-2 cells at a dose of 100 μg mL^−1^.^48^

Cheng *et al.* developed MSNs as nanocarrier for co-delivery of Cur and plasmid and then investigated the neuroprotective effects of this nanocarrier in order to promote neurite outgrowth. Cur acts as a natural antioxidant to protect neurons from ROS-induced damage, and the plasmid RhoG-DsRed contributes to neuroprotection by promoting neurite outgrowth through the formation of filopodia and lamellipodia. The complex of TAT peptide (transactivator of transcription) with plasmid RhoG-DsRed was employed as a noncovalent gatekeeper in this nanoformulation and the dissociation of this complex from the MSNs surface resulted in the release of Cur from the pores of MSNs. TAT peptide enhanced nuclear plasmid delivery of RhoG-DsRed in cells and thus increased gene expression.^55^

Recently, Cheng *et al.* designed and synthesized an asymmetric lollipop-like MSNs which composed of spherical Fe_3_O_4_-MSNs as core–shell and ethane bridged periodic mesoporous organosilica (EPMO) as nanorods tail. Cur and gentamicin sulfate were then loaded into the MSNs pores. The two independent spaces (hydrophilic and hydrophobic sites) in this nanocarrier make it suitable for encapsulation of both hydrophobic Cur and hydrophilic gentamicin sulfate. The findings of cell cytotoxicity in MCF-7 cells revealed that the Cur and gentamicin sulfate encapsulated into the lollipop-like MSNs had higher cytotoxicity (89.6%) than free Cur (48.2%) and gentamicin sulfate (19%) which can be attributed to the synergic effects of Cur and gentamicin sulfate. The antibacterial effects of this dual-drug nanocarrier were higher than free Cur, gentamicin sulfate and Cur or gentamicin sulfate-loaded MSNs.^66^

PEGylated lipid bilayer coated MSNs were employed as nanocarrier for co-delivery of paclitaxel and Cur and its anticancer effects were evaluated *in vivo* and *in vitro*. Compared to paclitaxel powder suspension, PEGylated lipid bilayer-MSNs improved paclitaxel solubility and offered sustained drug release. The co-delivery effects of paclitaxel and Cur on breast cancer decreased the dosage of drugs and its toxic side effects, while its anticancer effects were higher than free Cur and paclitaxel. Co-delivery of paclitaxel and Cur induced apoptosis and downregulated the expression of Bax in MCF-7 cell lines. The finding of *in vivo* study revealed that paclitaxel and Cur encapsulated MSNs could efficiently concentrate in the tumor site and diminish tumor size.^69,70^

The effect of co-delivery of Cur with siRNA (small interfering RNA) loaded-MSNs were evaluated on the cytokine release and its cytotoxicity was investigated on both macrophage cell line and on A549 cells as a model for alveolar epithelial cells. The cytotoxicity assay revealed no cytotoxicity for concentrations ranging from 10 to 100 μg mL^−1^ during a 48 hours incubation period. The co-delivery of Cur with siRNA encapsulated into the MSNs significantly inhibited the TNF-α release when compared to free Cur and siRNA, indicating that it might be a promising dual-drug delivery platform for lowering macrophage inflammatory responses in the lungs.^81^

Lojkowski *et al.* designed and synthesized phosphonate-functionalized MSNs using (3-(trihydroxysilyl) propyl methylphosphonate monosodium to improve the loading content of Cur and colchicine. After the synthesis of phosphonate-functionalized MSNs, Cur and colchicine or their combination were loaded on MSNs and then the core–shell structure was completed by coating with folic acid conjugated with chitosan-cellulose polymer. The anticancer effects of these nanoformulations were evaluated against various cell lines including HCT-116 colon cells, A549, HOS and MCF-7, in which co-delivery system of Cur and colchicine showed a strong synergistic effect and more anticancer activity against these cancer cell lines and particularly HCT-116 colon cells when compared with those of single delivery or free Cur and colchicine. Based on the findings, the expression of p53, caspase-3, and Bax was effectively increased, while the expression of Bcl-2 was inhibited in the presence of co-delivery system of Cur and colchicine. The lowest IC_50_ value for the co-delivery system of Cur and colchicine coated with a polymeric shell conjugated with Folic acid was 4.1 μg mL^−1^.^123^

Gallium is considered as an excellent potential candidate for cancer treatment, which effectively induces apoptosis by destroying the three-dimensional structure of DNA and inhibiting DNA polymerase, ATPase, and tyrosine-specific protein phosphatase activity.^124^ The cytotoxic effects of co-delivery of gallium(iii) nitrate and Cur loaded on amine functionalized-MSNs were evaluated against breast adenocarcinoma cells (MCF-7) and the IC_50_ value was 25 μM, which was significantly lower compared to incubation of MCF-7 cells with free gallium (40 μM). This co-delivery system induces apoptosis by upregulating caspases 9, 6, PARP, and GSK 3β and reducing the expression of mitochondrial proteins in MCF-7 cells.^125^

**Fig. 5:**
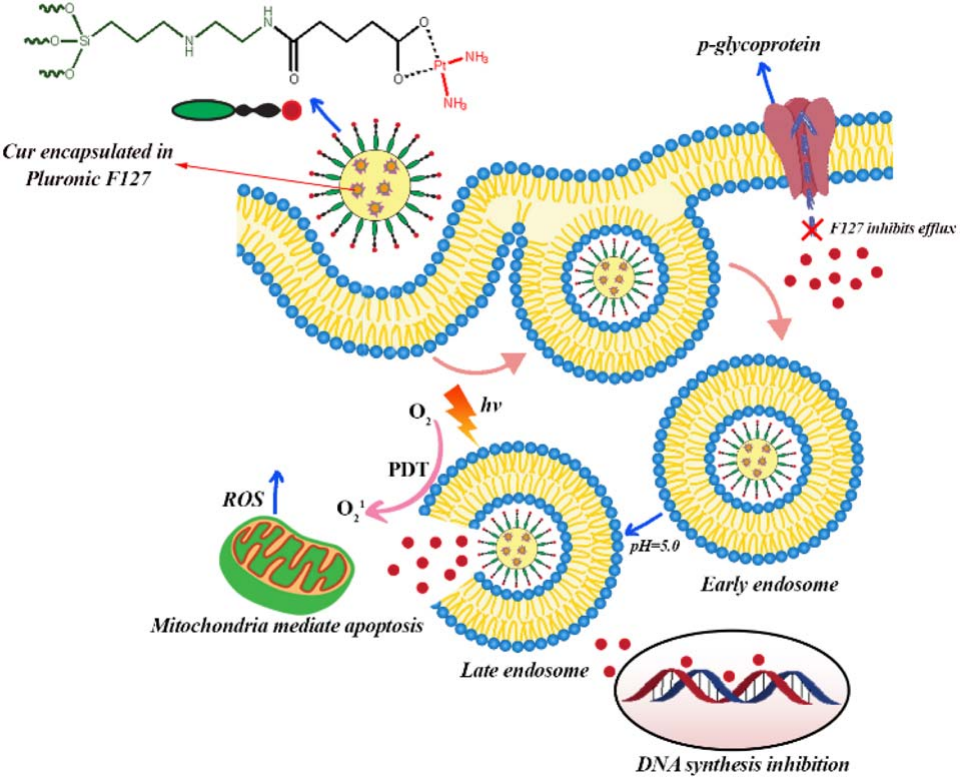
Cellular uptake of curcumin and cisplatin prodrug-encapsulated MSNs, pH-sensitive drug release, and the involvement in chemo- and photodynamic therapy in MES-SA/DX5 cells.

Carboxylation and carboxymethylation of the surface of MSNs can provide a wide range of derivatives and increase the stability of MSNs in aqueous solution at different pH ranges.^126,127^ Recently, Busa *et al.* investigated chemo and photodynamic therapy of Cur and cisplatin prodrug-encapsulated MSNs against multidrug resistance of MES-SA/DX5 cancer cells. In this study, cisplatin was immobilized on the surface of MSNs modified with carboxylate groups. For the synthesis of co-delivery system, Cur first was encapsulation in the Pluronic F127 micelle due to inhibitory effects of Pluronic F127 on p-glycoprotein (Fig. 5). Fluorocarbon surfactant was used as surfactant and by adding TEOS, Cur-embedded MSNs were obtained. In the next step, AAS as diamine silane agent and glutaric anhydride as carboxylic acid group agent were used to modify the surface of MSNs into amine- and carboxylic acid functionalized MSNs, respectively. Finally, diaqua-cisplatin is a highly reactive form of cisplatin that reacted with the carboxylic acid group on the surface of MSNs. The result of *in vitro* cellular uptake studies showed that co-delivery system of Cur and cisplatin was efficiently internalized by endocytosis and released both Cur and cisplatin into the cytosol. The level of ROS increased under light irradiation due to the presence of Cur as a photosensitizer in the MSNs. Therefore, the synergistic effects of chemo- and photodynamic therapies increased the anticancer activity of this co-delivery system against MES-SA/DX5 resistance cancer cells (Fig. 5).^127^

## Biological activity of Cur-loaded MSNs

Breast cancer is one of the most frequent cancers in women, and its prevalence has risen in recent years.^128^ Cur has been shown to suppress angiogenesis and metastasis in breast tumors.^129,130^ MSNs functionalized with folic acid or hyaluronic acid were employed as a multifunctional nanocarrier for targeted Cur delivery into breast cancer cells. The effects of free Cur, MSNs functionalized with hyaluronan, and polyethyleneimine-folic acid on MDA-MB-231 cell viability were 37, 16, and 14 μg mL^−1^, respectively. The greater cytotoxicity of MSNs modified with hyaluronan and polyethyleneimine-folic acid compared to free Cur may be attributed to their affinity for the folic acid receptor and CD44, both of which target endocytosis.^52^ According to the findings, targeted delivery of Cur-loaded MSNs *via* hyaluronic acid in breast cancer cells accelerated apoptosis *via* the NF-κB and Bax pathways, increased intracellular ROS production and cell cycle arrest at G2/M phase, inhibited cell migration, increased cellular uptake of Cur in tumor tissue, and decreased tumor volume in tumor-bearing mice.^67^

**Fig. 6:**
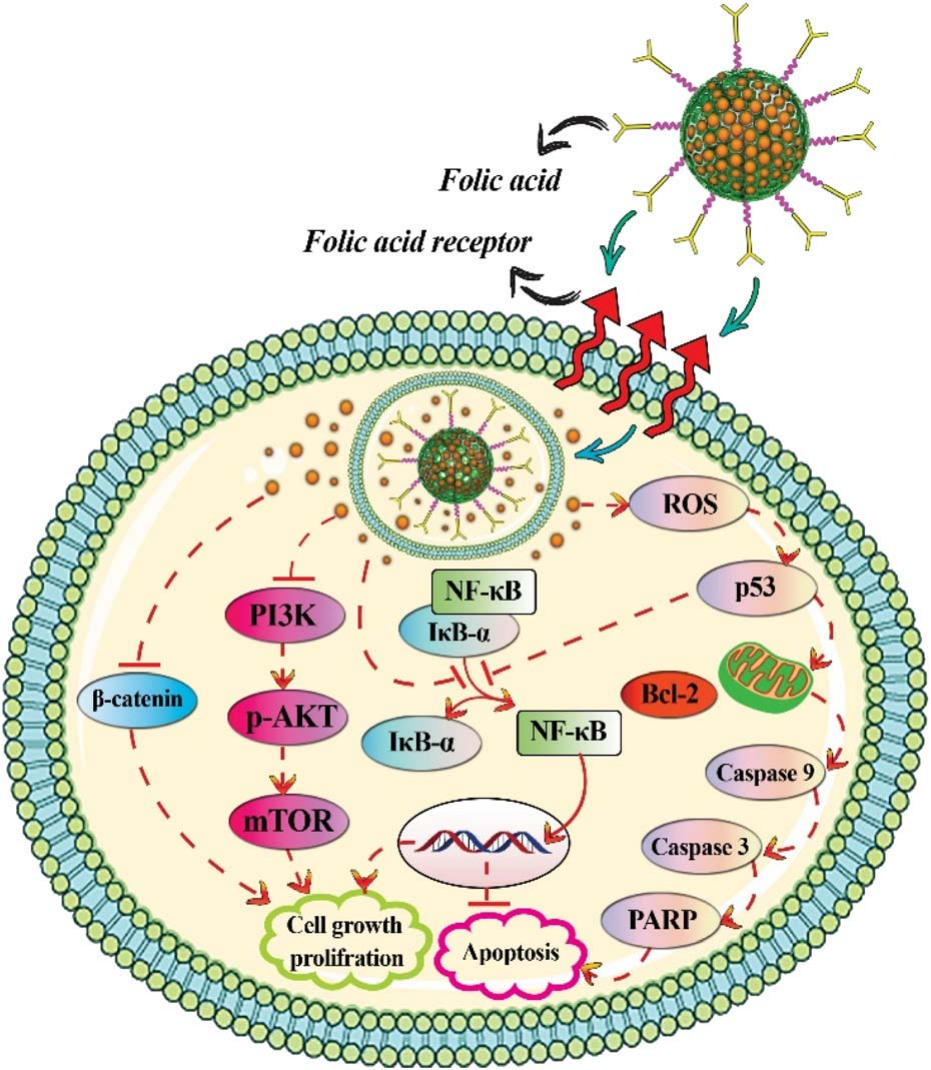
Anti-breast cancer activities of curcumin-loaded mesoporous silica nanoparticles.

Cur-loaded and calcium-doped dendritic MSNs modified with folic acid as pH-responsive and targeted drug delivery system effectively inhibited proliferation of human breast cancer MCF-7 cells. Cur-loaded dendritic MSNs had greater anticancer efficacy than free Cur, due to increased solubility and bioavailability, reduction of PI3K/AKT/mTOR and Wnt/β-catenin signaling, and the activation of mitochondria-mediated apoptotic pathway (Fig. 6).^54^ Free Cur, Cur loaded-MSNs immobilized with Au nanoparticles, and Cur loaded-MSNs immobilized with Au nanoparticles and modified with folic acid had IC_50_ values of 53.7, 48.93, and 21.20 μg mL^−1^, respectively, against MCF-7 cancer cell lines. The IC_50_ values against A549 cell lines were 88.53, 54.67 and 18.21 μg mL^−1^, respectively.^58^

The cytotoxicity of guanidine functionalized-PEGylated MSNs were evaluated against breast adenocarcinoma cells (MCF-7), mouse breast cancer cells (4T1), and human mammary epithelial cells (MCF10A) and the findings revealed that the IC_50_ values for MCF7 and 4T1cell lines were 19.5 and 14.8 μM, respectively, within 48 hours. However, it had no effect on MCF10A (a non-tumorigenic epithelial cell line) cell proliferation. The pure nanocarrier had no cytotoxicity against all three cell lines even at concentration of 60 μM.^68,72^ Viswanathan *et al.* also reported that guanidine–Cur complex-loaded amine-functionalized MSNs effectively showed anti-breast cancer activity against MCF-7 cells through downregulation of phosphorylation in Ser471 of Akt, Ser259 of c-Raf, and Ser241 of PDK1 as well as upregulation of phosphorylation in GSK-3β Ser9, cleaved caspases, and cleaved PARP. Based on docking studies, guanidine-Cur complex clearly targets tumor-suppressing proteins in MCF-7 cells through hydrogen bonding and electrostatic interactions with lower binding affinity, leading to apoptosis.^131^

Recently, the antimicrobial effects of Cur-loaded MSNs were evaluated against clinically isolated *Porphyromonas gingivalis*, which is the main cause of periodontal disease. According to the findings, *P. gingivalis* was sensitive to the Cur-loaded MSNs at concentrations of 50–6.25 μg mL^−1^ with the greatest inhibition zone (*p* ≤ 0.05) at the concentration of 50 μg mL^−1^.^132^

Harini *et al.* investigated the mechanism of Cur delivery from functionalized MSNs with polyethyleneimine and the signaling proteins that were triggered to induce apoptosis against MCF-7 cells. They found that Cur induces apoptosis through the activation of caspase 9, poly (ADP-ribose) polymerase (PARP) (Fig. 6), C/EBP homologous protein (CHOP) and phosphatase and tensin homolog (PTEN) and downregulation of Akt1. According to the results of electron microscopic analysis, Cur loaded into functionalized MSNs caused apoptosis by disrupting mitochondria and nucleus and altering the inner mitochondrial membrane from a normal vesicular structure to swollen mitochondria after 48 hours. In contrast, free Cur induced apoptosis in MCF-7 cells by destroying the chromosome and the plasma membrane. In fact, the treatment with Cur loaded into functionalized MSNs cause mitochondrial disruption, which leads to increased expression of cleaved caspase 9 and caspase 12 when compared to free Cur treatment.^80^

Various studies have shown that Cur has hepatoprotective effects.^133–135^ Recently, the antitumor activities of Cur loaded-PEGylated MSNs and free Cur were studied on HepG2 cells. The cytotoxicity results showed that Cur loaded-PEGylated MSNs (Cur concentration = 36 μg mL^−1^) had cell viability of less than 10% and about 7% after 24 and 48 hours, respectively, whereas HepG2 cells treated with free Cur had cell viability of less than 76% after 48 hours at the same concentration of Cur (Cur concentration = 36 μg mL^−1^).^76^ PEGylation of MSNs, which contributes to increase in cellular uptake and intracellular accumulation of Cur loaded MSNs within tumor cells, is responsible for the considerable cytotoxic activity of Cur loaded-PEGylated MSNs (IC_50_ = 20 μg mL^−1^).^75^

Kong *et al.* investigated the cytotoxicity effects of Cur encapsulated into the MSNs and Cur encapsulated into the MSNs modified with chitosan on hepatocellular carcinoma cells. Findings revealed that modification of MSNs with chitosan increased Cur stability and cytotoxicity against hepatocellular carcinoma cells. Encapsulation of Cur into MSNs pores improved antioxidant activity, biocompatibility and anticancer activity.^64^

The hepatoprotective effects of Cur-loaded MSNs were investigated on CCl_4_-induced hepatotoxicity Wistar rats. The results demonstrated a significant decrease in ALT and AST compared to the control group 14 days after treatment, but histological examination revealed a higher number of necrotic hepatic cells in the Cur-loaded MSNs group (10 mg kg^−1^ b.w.) than in the free Cur group (2 mg kg^−1^ b.w.). The author of this study didn't explore the effect of MSNs in a separate group, thus it's unclear whether the increased number of necrotic hepatic cells in the Cur loaded-MSNs group was due to MSN toxicity or the high concentration of Cur.^63^

Cur has been shown to have cardioprotective effects in rats with myocardial necrosis *via* inhibiting free radical production in rats with myocardial ischemia.^136^ Yadav *et al.* investigated the cardioprotective effects of free Cur and Cur loaded-MSNs against doxorubicin-induced myocardial toxicity in rats. For two weeks, a similar concentration of Cur was given to each group as pretreatment, and then treatment was continued for another two weeks with doxorubicin. Both free Cur and Cur loaded-MSNs significantly reduced malondialdehyde levels while also increasing antioxidant enzymes including GSH, SOD, and CAT when compared to the doxorubicin-treated group. According to the findings, the efficacy of Cur loaded-MSNs was greater than that of free Cur, due to the improved bioavailability and solubility of Cur encapsulated into MSNs pores than free Cur. Histopathological findings revealed that Cur loaded-MSNs-treated group had less loss of myofibrils and vacuolization of the cytoplasm than the doxorubicin-treated group.^50^

Ribeiro *et al.* used the combination of chitosan (1% w/v) and poloxamer 407 (18% w/v) as a thermo-responsive hydrogel to facilitate the intranasal administration of Cur-loaded MSNs in a STZ-induced Alzheimer's disease model. The results of cytotoxicity assay showed that the combination of Cur-loaded MSNs with thermo-responsive hydrogel was safe and biocompatible for L929 cells line at the concentration of 50 μg mL^−1^.

The ex vivo permeation studies of Cur loaded-MSNs and their combination with thermo-responsive hydrogel showed higher permeation values of thermo-responsive hydrogel formulation (28.40 μg cm^−2^) than Cur loaded-MSNs (12.46 μg cm^−2^). Furthermore, the intranasal administration of thermo-responsive hydrogel-Cur loaded MSNs effectively increased memory retention and improved cognitive deficit in Alzheimer's disease mice.^137^

Cur loaded-MSNs demonstrated effective anticancer activity against HN5 cell line (head and neck cancer cells), and the cytotoxic effects of Cur loaded-MSNs was greater than those of free Cur treatment. Incubation of HN5 cells with Cur loaded-MSNs significantly reduced Bcl-2 expression, while Bax/Bcl-2 ratio was increased by 3.43-fold compared to the untreated HN5 cells.^138^

## Limitations of current research and future perspectives

Some of the current studies suggested that functionalized MSNs as tumor-targeting Cur carriers, can synergistically increase the cytotoxic effects of Cur on cancer cells. However, more detailed studies are needed to prove this claim. Most of studies used surfactants such as CTAB to prepare MSNs. Therefore, the results of cytotoxicity assay may be influenced by the presence of surfactant impurity or other impurities introduced during the functionalization process of MSNs. Therefore, the purity of MSNs and functionalized MSNs should be investigated in details. Cur-loaded MSNs can be administered in a variety of ways that may alter the results of the biological assay. Therefore, it is necessary to focus on comparing different methods of Cur-loaded MSNs administration. Although the surface modification of MSNs provides strategies to produce more effective nanocarriers with fewer adverse effects for humans, a thorough understanding of the behavior of functionalized MSNs in the human body is important to maximize their therapeutic potential in targeted drug delivery. Based on current studies, loaded MSNs have promising potential for therapeutic applications, however, more preclinical studies are needed to justify further clinical studies.

## Conclusions

In this review, we summarized the latest progress in the different synthesis methods of Cur-loaded MSNs and their potential applications. The use of Cur-loaded MSNs allows for targeted drug delivery to specific cells and tissues, potentially overcoming the limitations of traditional chemotherapy. MSNs also enable co-delivery of Cur with other anticancer drugs, increasing the cytotoxic activity of the treatment. However, while Cur-loaded MSNs show promise for therapeutic applications, further studies are needed to assess their clinical efficacy and commercial viability. Additionally, it is important to fully understand the behavior of MSNs in the human body to maximize their therapeutic potential.

## Author Contributions

Milad Iranshahy: searching and collecting research articles, revising and writing the manuscript. Mohammad Yahya Hanafi-Bojd: main idea of the article. Seyed Mohammad Nabavi: editing the article. Satar Saberi: searching and collecting research articles. Rosanna Filosa: writing the manuscript. Iman Farzam Nezhad: searching and collecting research articles. Maede Hasanpour: idea conceptualization, revising and writing the manuscript.

## Conflicts of interest

There are no conflicts to declare.

## Supplementary Material
